# Evaluation of performance and perceived utility of mental healthcare indicators in routine health information systems in five low- and middle-income countries

**DOI:** 10.1192/bjo.2019.22

**Published:** 2019-08-06

**Authors:** Mark Jordans, Dan Chisholm, Maya Semrau, Dristy Gurung, Jibril Abdulmalik, Shalini Ahuja, James Mugisha, Ntokozo Mntambo, Fred Kigozi, Inge Petersen, Rahul Shidhaye, Nawaraj Upadhaya, Crick Lund, Graham Thornicroft, Oye Gureje

**Affiliations:** Reader, Centre for Global Mental Health, Institute of Psychiatry, Psychology and Neuroscience, King's College London, UK; Programme Manager for Mental Health, Regional Office for Europe, World Health Organization, Switzerland; Research Fellow, Centre for Global Mental Health, Institute of Psychiatry, Psychology and Neuroscience, King's College London; and Global Health and Infection Department, Brighton & Sussex Medical School, Brighton, UK; Research Coordinator, Transcultural Psychosocial Organization, Nepal; Senior Lecturer, Department of Psychiatry, University of Ibadan, Nigeria; Researcher, Public Health Foundation of India & Centre for Global Mental Health, Institute of Psychiatry, Psychology and Neuroscience, King's College London, UK; Senior Lecturer, Kyambogo University, Kampala, Uganda & Butabika National Referral and Teaching Mental Hospital, Uganda; Researcher, University of Kwazulu-Natal, South Africa; Senior Consultant Psychiatrist and Researcher, Butabika National Referral and Teaching Mental Hospital, Uganda; Research Professor and Director, Centre for Rural Health, School of Nursing and Public Health, University of KwaZulu-Natal, South Africa; Clinical Psychiatrist, Public Health Foundation of India, India; and CAPHRI School for Public Health and Primary Care, Maastricht University, the Netherlands; Researcher, Transcultural Psychosocial Organization, Nepal; Professor of Public Mental Health and Professor of Global Mental Health and Development, Alan J Flisher Centre for Public Mental Health, Department of Psychiatry and Mental Health, University of Cape Town, South Africa, and Centre for Global Mental Health, Health Service and Population Research Department, Institute of Psychiatry, Psychology and Neuroscience, King's College London, UK; Professor of Community Psychiatry, Centre for Global Mental Health and Centre for Implementation Science, Institute of Psychiatry, Psychology and Neuroscience, King's College London, UK; Professor of Psychiatry and Director, WHO Collaborating Centre for Research and Training in Mental Health, Neurosciences and Substance Abuse, Department of Psychiatry, University of Ibadan, Nigeria; and Professor Extraordinary, Department of Psychiatry, Stellenbosch University, South Africa

**Keywords:** Mental healthcare, indicators, primary healthcare, low- and middle-income settings, health information system

## Abstract

**Background:**

In most low- and middle-income countries (LMIC), routine mental health information is unavailable or unreliable, making monitoring of mental healthcare coverage difficult. This study aims to evaluate a new set of mental health indicators introduced in primary healthcare settings in five LMIC.

**Method:**

A survey was conducted among primary healthcare workers (*n* = 272) to assess the acceptability and feasibility of eight new indicators monitoring mental healthcare needs, utilisation, quality and payments. Also, primary health facility case records (*n* = 583) were reviewed by trained research assistants to assess the level of completion (yes/no) for each of the indicators and subsequently the level of correctness of completion (correct/incorrect – with incorrect defined as illogical, missing or illegible information) of the indicators used by health workers. Assessments were conducted within 1 month of the introduction of the indicators, as well as 6–9 months afterwards.

**Results:**

Across both time points and across all indicators, 78% of the measurements of indicators were complete. Among the best performing indicators (diagnosis, severity and treatment), this was significantly higher. With regards to correctness, 87% of all completed indicators were correctly completed. There was a trend towards improvement over time. Health workers' perceptions on feasibility and utility, across sites and over time, indicated a positive attitude in 81% of all measurements.

**Conclusion:**

This study demonstrates high levels of performance and perceived utility for a set of indicators that could ultimately be used to monitor coverage of mental healthcare in primary healthcare settings in LMIC. We recommend that these indicators are incorporated into existing health information systems and adopted within the World Health Organization Mental Health Gap Action Programme implementation strategy.

**Declaration of interest:**

None.

Globally, and especially in low- and middle-income countries (LMIC), there is a large treatment gap for mental disorders, as indicated by the lack of service for a great majority of people in need of mental healthcare.^1^ Integrating mental healthcare into primary healthcare has been recommended as a way of bridging this treatment gap by offering more accessible, holistic and less stigmatising services to people in need.^2^ The World Health Organization (WHO) launched the Mental Health Gap Action Programme Intervention Guide (mhGAP-IG) in 2010 (and a second version in 2016), to provide evidence-based clinical guidance for the assessment and management of priority mental, neurological and substance use disorders in LMIC non-specialist settings.^3^ Building on this development, and in order to facilitate the process of integration, a broader health system strengthening approach involving policy changes, sustainable financing mechanisms and workforce development is required.

One such system requirement is a well-functioning routine health information system that ensures the production, analysis, dissemination and use of reliable and timely information related to service delivery.^4^ Moreover, in order for the much-needed investments in scaling up of mental health services to be efficiently used, comprehensive and accurate information on the availability, utilisation and equity of such services is required. However, health information systems to support actions in mental healthcare are weak in LMIC. Most routine information systems do not have any, or have very limited, indicators related to mental healthcare. When information is collected, it often produces unreliable data.^5^ The aim of this study was to evaluate the performance and perceived utility of a new set of indicators for routine monitoring of mental healthcare within primary healthcare settings in five LMIC.

## Method

### Setting

The study was conducted in five LMIC in Africa and Asia participating in the Emerald programme (India, Nepal, Nigeria, South Africa and Uganda). The Emerald program aimed to improve mental health outcomes by generating evidence and capacity to enhance health system performance in LMIC.^6^

### Emerald programme

As a way of responding to the lack of indicators to monitor mental healthcare, the Emerald programme developed, through a Delphi study, a set of indicators that can be used to appraise mental healthcare needs, utilisation, outcomes and costs.^7^ From an initial set of 52 generated items, the eight most highly prioritised indicators were selected and operationalised through consultative workshops in each country setting convened to develop data collection formats suitable for use by frontline health workers. The eight indicators included were: diagnosis (as multiple-choice response options, which included depression, alcohol use disorder, schizophrenia and epilepsy, as well as other disorders depending on priorities per country team), exact diagnosis (as open response format), severity, functioning, administered treatment, referral, follow-up and payment for services (the latter six items all had multiple response options, including none or not relevant). These formats were subsequently integrated into district-level mental healthcare programmes, as part of ongoing research programmes evaluating the integration of mental healthcare into primary healthcare.^8,9^ (See supplementary File 1, available at https://doi.org/10.1192/bjp.2019.22, for the standard form, which was adapted in each setting.)

### Instruments

First, a structured questionnaire was developed for the purpose of this study, to assess health workers' perceptions of the utility (i.e. acceptability and feasibility) of using the data collection format for the new indicators. The questionnaire consisted of 14 questions, which were administered through interviews conducted by trained research assistants. Second, a health information case record review form was developed to assess the performance of the newly introduced indicators based on observation of the completed formats. A trained research assistant checked all case records, first assessing whether the completion of each of the indicators (i.e. level of completion (response options: ‘yes/no’)). Subsequently, for each completed indicator, the research assistant assessed the level to which completed information was correctly or incorrectly completed (i.e. level of correctness of completion (response options: ‘correct/incorrect’), with the latter defined as ‘illogical completion/missing information/illegible information’). The form consisted of eight items and the ratings of its completion and correctness of completion were made by trained research assistants.

### Sample

See Table 1 for an overview of the number of interviews and case record reviews across sites and time-points. The structured interviews were administered to 272 health workers who had been trained to provide services and to use the new data collection forms. The sample was selected from primary healthcare facilities (all countries) and a few district hospitals (India and Uganda). Selection was done either randomly (in Nepal, among the 205 trained health workers, 177 were still in post, among whom 50 were randomly selected; in Nigeria, two health workers were randomly selected among the 4–6 trained health workers per facility) or purposively (in South Africa, staff were selected based on availability and willingness until the target sample was reached; in Uganda and India, the health facility in charge approached health workers involved in record keeping or mental health services, respectively). The health information records (*n* = 586) assessed for completion and correctness were selected randomly in all of the countries. Randomisation of the case records was done by: (a) randomly selecting a specified number of facilities and (b) randomly selecting a number of case records within the selected facility.

**Table 1:** Overview of sample

	India (*N*)	Nepal (*N*)	Nigeria (*N*)	South Africa (*N*)	Uganda (*N*)	Total
T1 surveys of health workers	16	50	36	24	26	152
T2 surveys of health workers	9	45	36	18	12	120
T1 record reviews	61	95	45	32	66	299
T2 record reviews	74	58	43	40	69	284

T1 refers to 2–4 weeks after introduction of the new indicators within primary healthcare settings; T2 refers to 6–9 months after the introduction.

### Procedure

The interviews with health workers and the health information record reviews were performed 2–4 weeks after the new forms were introduced and had started to be used in the clinics. We chose the period of 2–4 weeks to allow some time for proper adoption of the procedure post training, including the printing and distribution of the required registers/forms. This time frame was to allow the staff some time to establish familiarity with the new forms before the interviews/assessments were conducted at baseline. A second wave of interviews and record reviews were conducted with the same health workers and in the same health facilities 6–9 months after the introduction of the forms, to assess whether there was improvement, deterioration or constancy in the scores over time. Interviews and record reviews were conducted by research assistants who had received an average of 5 days training at each of the participating sites.

### Data analysis and management

Data from questionnaires and record review forms (for both time points; T1 and T2) were entered into SPSS and analysed using descriptive statistics. The calculation of the proportion of completion of indicators was based on the total number of cases; correctness was calculated based on the completed indicators and was determined by whether the information entered in the data form was assessed by the research assistant to be correct. Thus, for example, if the indicator slot for ‘diagnosis’ was filled, a rating was made to indicate that the information was indeed collected (completion) and whether the information filled into the slot was indeed a relevant diagnosis (correctness). Data collection took place between August 2015 and October 2017.

### Ethics

Ethical approval for this study was obtained from King's College London, the World Health Organization and the institutional review boards of each of the participating sites. Subsequently, permission was obtained from the appropriate Department/Ministry of Health officials, as well as from the healthcare workers and health facility managers. Written informed consent was sought and obtained from all study participants. The facility managers were approached by research assistants for their participation in the study and for permission to access the health information records.

## Results

Across the five sites, 272 health workers were interviewed and 586 case records were reviewed. Table 2 shows the performance ratings of the new indicators with regard to levels of completion and correctness of completion. Overall, high levels of completion and of correctness were achieved for most of the indicators at both time points. The combined completion and correctness ratings for both time points were especially high for diagnosis (94 and 95%, respectively), severity (83 and 99%, respectively) and treatment (92 and 86%, respectively). These scores were slightly lower for exact diagnosis (72 and 84%, respectively), functioning (76 and 83%, respectively) and follow-up (83 and 82%, respectively). To give an indication of the samples of cases for which records were reviewed, the breakdown of diagnoses recorded was as follows (for T1 data): AUD (18% in Nepal, 3% in Uganda, 20% in India, 22% in Nigeria); depression (28% in Nepal, 100% in South Africa, 14% in Uganda, 46% in India, 60% in Nigeria); epilepsy (13% in Nepal, 68% in Uganda, 5% in Nigeria); psychosis (32% in Nepal, 5% in Uganda, 1% in India, 13% in Nigeria); and others (such as anxiety disorders, dementia, development disorders; 9% in Nepal, 10% in Uganda, 33% in India).

**Table 2:** Levels of completion and correctness of completion of mental health indicators in routine heath information systems across five LMICs

			Diagnosis *N* (%)	Exact diagnosis *N* (%)	Severity *N* (%)	Functioning *N* (%)	Treatment administered *N* (%)	Referral *N* (%)	Follow-up *N* (%)	Payment *N* (%)
India	Completion	T1 *N* = 61	61 (100)	49 (80.0)	61 (100)	n/a	60 (98.0)	2 (100)^a^	41 (100)^a^	n/a
		T2 *N* = 74	65 (87.8)	63 (85.1)	29 (39.1)	n/a	65 (87.8)	4 (100)^a^	3 (100)^a^	n/a
	Correctness^b^	T1	60 (98.0)	46 (94.0)	60 (98.0)	n/a	42 (70.0)	2 (100)^a^	29 (100)^a^	n/a
		T2	64 (98.4)	55 (87.3)	29 (100)	n/a	64 (98.4)	4 (100)^a^	3 (100)^a^	n/a
Nepal	Completion	T1 *N* = 95	82 (86.3)	27 (28.4)	81 (85.3)	45 (47.4)	76 (80.0)	16 (16.8)	55 (57.9)	35 (36.8)
		T2 *N* = 58	57 (98.3)	43 (74.1)	56 (96.6)	38 (65.5)	55 (94.8)	32 (55.2)	51 (87.9)	37 (63.8)
	Correctness^b^	T1	79 (96.3)	24 (88.9)	81 (100)	28 (62.2)	71 (93.4)	15 (93.7)	52 (94.5)	29 (82.8)
		T2	57 (100)	43 (100)	56 (100)	38 (100)	55 (100)	32 (100)	51 (100)	37 (100)
Nigeria	Completion	T1 *N* = 45	36 (80)	n/a	34 (75.6)	34 (75.6)	45 (100)	45 (100)	27 (60)	39 (86.7)
		T2 *N* = 43	43 (100)	n/a	42 (97.7)	37 (86.0)	42 (97.7)	42 (97.7)	43 (100)	43 (100)
	Correctness^b^	T1	25 (69.4)	n/a	32 (94.1)	32 (94.1)	44 (97.8)	42 (93.3)	22 (81.5)	39 (100)
		T2	43 (100)	n/a	41 (97.6)	36 (97.3)	41 (97.6)	39 (92.9)	43 (100)	43 (100)
South Africa	Completion	T1 *N* = 32	28 (87.5)	31 (96.9)	30 (93.8)	n/a	20 (62.5)	31 (96.9)	32 (100)	n/a
		T2 *N* = 40	40 (100)	40 (100)	40 (100)	n/a	40 (100)	40 (100)	40 (100)	n/a
	Correctness^b^	T1	28 (100)	31 (100)	30 (100)	n/a	20 (100)	31 (100)	31 (96.9)	n/a
		T2	40 (100)	40 (100)	40 (100)	n/a	40 (100)	40 (100)	40 (100)	n/a
Uganda	Completion	T1 *N* = 66	65 (98.5)	43 (65.2)	n/a	64 (97.0)	62 (95.4)	22 (33.9)	59 (89.4)	42 (63.6)
		T2 *N* = 69	69 (100)	62 (89.9)	n/a	68 (98.6)	69 (100)	27 (39.1)	63 (91.3)	22 (31.9)
	Correctness^b^	T1	62 (95.4)	23 (53.5)	n/a	58 (95.1)	32 (50.0)	7 (35.0)	18 (31.0)	0
		T2	61 (88.4)	37 (59.7)	n/a	45 (66.2)	52 (75.4)	9 (33.3)	45 (71.4)	8 (36.4)

a. The response options and the denominators for these items were different from the other countries; these are therefore not included in the summaries of results.

b. Correctness refers to level of completed indicators that was correctly completed (as opposed to ‘incorrect completion’).

The combined completion and correctness for both time points was lowest for referral (60 and 84%, respectively) and payment (58 and 72%, respectively). The lower scores for referral and payments were all clustered within one country setting (i.e. Uganda), where 7/8 measurements points showed results that were <50% positive. The only other trend for such lower scores were for the completion of indicators at T1 in Nepal (4/8 scoring 50% positive). In South Africa scores for completion and correctness were high across indicators and time points with 18/24 measurements showing 100% positives. Furthermore, the T2 correctness measurements for Nepal and Nigeria were all over 90%. These contributed to a cross-country trend for improvement of scores between T1 and T2 for completion of indicators (7/8 showed improvement over time) and for correctness of completion (6/8 showed improvement). There was only a cross-country trend for deterioration over time for completion of the severity indicator.

The utility of the new mental health indicators was assessed through health workers' perceptions of the feasibility and acceptability of using the indicators in routine healthcare settings across the five countries (Table 3). Overall, there were moderate-to-high scores on perceived utility. All but two items had over 80% positive responses (positive responses were defined as the combination of ‘agree’ and ‘strongly agree’) across time and countries. These were especially high for perceived importance of routine mental health indicators (96% positive) motivation to use these indicators (95%), as well as perceived relevance (91%), integration in health workers' daily work (90%), ease of use (86%), confidence to use (84%) and overall satisfaction with the new forms (82%). Perceived usefulness of the indicators in daily practice fell a bit behind, with 74% positive responses. Having insufficient time was the only item where only a minority had a positive response (33%). Indeed, a combined average of 27% of the reports showed that more than 10 min additional time was spent on recording.

**Table 3:** Health workers' perceptions on using mental health indicators in the routine health information system across five LMICs

		India		Nepal		Nigeria		South Africa		Uganda	
		T1 *N* = 16	T2 *N* = 9	T1 *N* = 50	T2 *N* = 45	T1 *N* = 36	T2 *N* = 36	T1 *N* = 24	T2 *N* = 18	T1 *N* = 26	T2 *N* = 12
Item	Response	*N* (%)	*N* (%)	*N* (%)	*N* (%)	*N* (%)	*N* (%)	*N* (%)	*N* (%)	*N* (%)	*N* (%)
Currently involved in routine data collection?	Yes	9	7 (77.8)	n/a	n/a	36 (100)	36 (100)	21 (87.5)	13 (72.2)	n/a	n/a
		7	2 (22.2)			0	0	3 (12.5)	5 (27.8)		
Received training on routine data collection?	Yes	16	9 (100)	43 (86.0)	43 (95.6)	18 (50)	32 (88.9)	8 (33.3)	7 (38.9)	9 (34.6)	5 (41.7)
	No	0	0	7 (14.0)	2 (4. 4)	18 (50)	4 (11.1)	16 (66.7)	11 (61.1)	17 (65.4)	7 (58.3)
How many mental illness patients do you see on average in a week?	1 or fewer	3 (18.8)	3 (42.9)	35 (70.0)	18 (40.0)	31 (86.1)	34 (94.4)	7 (29.2)	5 (27.8)	3 (11.5)	0
	2–4 per week	8 (50.0)	3 (42.9)	12 (24.0)	23 (51.1)	5 (13.9)	2 (5.6)	7 (29.2)	10 (55.6)	14 (53.9)	8 (66.7)
	5–7 per week	3 (18.8)	0	2 (4.0)	2 (4.4)	0	0	2 (8.3)	2 (11.1)	4 (15.4)	4 (33.3)
	8–10 per week	1 (6.3)	0	0	1 (2.2)	0	0	4 (16.7)	0	3 (11.5)	0
	>10 per week	1 (6)	1 (14.3)	1 (2.0)	1 (2.2)	0	0	4 (16.7)	1 (5.6)	2 (7.7)	0
How much time do you think you spend, on average, with each patient who has mental health problems?	<5 min	0	1 (14.3)	0	0	7 (19.4)	14 (38.9)	0	0	3 (11.5)	0
	5–10 min	2 (12.5)	1 (14.3)	6 (12.0)	12 (26.7)	9 (25)	7 (19.4)	4 (16.7)	2 (11.1)	10 (38.5)	4 (33.3)
	11–20 min	0	1 (14.3)	18 (36.0)	16 (35.6)	6 (16.7)	6 (16.7)	10 (41.7)	5 (27.8)	10 (38.5)	5 (41.7)
	21–30 min	2 (12.5)	0	13 (26.0)	10 (22.2)	7 (19.4)	5 (13.8)	9 (37.5)	7 (38.9)	2 (7.7)	3 (25)
	>30 min	12 (75.0)	4 (57.1)	13 (26.0)	7 (15.6)	7 (19.4)	4 (11.1)	1 (4.2)	4 (22.2)	1 (3.9)	0
How much time do you spend on average recording information for *any* patient?	<5 min	1 (6.3)	0	21 (42.0)	24 (53.3)	10 (27.8)	11 (30.6)	0	1 (5.6)	7 (28.0)	5 (41.7)
	5–10 min	2 (12.5)	4 (57.1)	26 (52.0)	15 (33.3)	16 (44.4)	14 (38.9)	7 (29.2)	9 (50.0)	14 (56)	5 (41.7)
	11–20 min	7 (43.8)	1 (14.3)	3 (6.0)	5 (11.1)	9 (25)	7 (19.4)	13 (54.2)	5 (27.8)	3 (12.0)	2 (16.7)
	>20 min	6 (37.5)	2 (28.6)	0	1 (2.2)	1 (2.8)	4 (11.1)	4 (16.7)	3 (16.7)	1 (4.0)	0
How much *additional* time was needed using the new format?	<5 min	0	1 (14.3)	13 (26.0)	8 (17.8)	13 (36.1)	9 (25)	12 (50.0)	2 (11.1)	14 (53.9)	4 (33.3)
	5–10 min	6 (37.5)	4 (57.1)	24 (48.0)	30 (66.7)	8 (22.2)	15 (41.7)	11 (45.8)	11 (61.1)	10 (38.5)	4 (33.3)
	11–20 min	3 (18.8)	0	10 (20.0)	5 (11.1)	6 (16.7)	8 (22.2)	0	1 (5.6)	2 (7.7)	3 (25.0)
	>20 min	7 (43.8)	2 (28.6)	3 (6.0)	2 (4.4)	9 (25)	4 (11.1)	1 (4.2)	4 (22.2)	0	1 (8.3)
How satisfied are you with the new format?	Very dissatisfied	0	0	1 (2.0)	1 (2.2)	1 (2.8)	1 (2.8)	0	1 (5.6)	2 (7.7)	0
	A little dissatisfied	1 (6.3)	2 (28.6)	1 (2.0)	4 (8.9)	0	1 (2.8)	0	1 (5.6)	1 (3.9)	2 (16.7)
	Neutral	3 (18.8)	0	1 (2.0)	0 (0.0)	2 (5.6)	8 (22.2)	1 (4.2)	7 (38.8)	4 (15.4)	0
	A little satisfied	8 (50.0)	2 (28.6)	36 (72.2)	28 (62.2)	11 (30.6)	3 (8.3)	5 (20.8)	4 (22.2)	11 (42.3)	5 (41.7)
	Very satisfied	4 (25.0)	3 (42.9)	11 (22.0)	12 (26.7)	22 (61.1)	23 (63.9)	18 (75.0)	5 (27.8)	8 (30.8)	5 (41.7)
It is possible to use the mental health indicators in routine practice	Strongly disagree	0	0	4 (8.0)	2 (4.4)	1 (2.8)	2 (5.6)	0	2 (11.1)	0	0
	Disagree	1 (6.3)	4(44.4	14 (28.0)	3 (6.7)	3 (8.3)	9 (25)	3 (12.5)	0	4 (15.4)	4 (33.3)
	Neutral	0	0	0	3 (6.7)	2 (5.6)	5 (13.9)	0	5 (27.8)	1 (3.9)	0
	Agree	8 (50.0)	3 (33.3)	27 (54.0)	29 (64.4)	18 (50)	15 (41.6)	20 (83.3)	6 (33.3)	19 (73.1)	5 (41.7)
	Strongly agree	7 (43.8)	2 (22.2)	5 (10.0)	8 (17.8)	12 (33.3)	5 (13.9)	1 (4.2)	5 (27.8)	2 (7.7)	3 (25.0)
I do not have enough time to complete additional questions for routine data collection	Strongly disagree	0	0	8 (16.0)	12 (26.7)	10 (27.8)	4 (11.1)	1 (4.2)	3 (16.7)	4 (15.4)	3 (25.0)
	Disagree	1 (96.3)	3 (42.9)	24 (48.0)	20 (44.4)	17 (47.2)	21 (58.3)	13 (54.2)	3 (16.7)	16 (61.5)	4 (33.3)
	Neutral	2 (12.5)	1 (14.3)	1 (2.0)	3 (6.7)	1 (2.8)	0	1 (4.2)	4 (22.2)	1 (3.9)	0
	Agree	10 (62.5)	3 (42.9)	16 (32.0)	7 (15.6)	7 (19.4)	10 (27.8)	9 (37.5)	7 (38.9)	4 (15.4)	5 (41.7)
	Strongly agree	3 (18.8)	0	1 (2.0)	3 (6.7)	1 (2.8)	1 (2.8)	0	1 (5.6)	1 (3.9)	0
Routine mental health data collection is important	Strongly disagree	0	0	0	0	1 (2.8)	1 (2.8)	0	0	0	2 (16.7)
	Disagree	0	1 (11.1)	1 (2.0)	0	1 (2.8)	0	0	0	0	0
	Neutral	0	1 (11.1)	0	0	1 (2.8)	1 (2.8)	0	1 (5.6)	0	0
	Agree	1 (6.3)	6 (66.7)	15 (30.0)	20 (44.4)	23 (63.9)	16 (44.4)	14 (58.3)	10 (55.6)	12 (46.2) 14	4 (33.3)
	Strongly agree	15 (93.8)	1 (11.1)	34 (68.0)	25 (55.6)	10 (27.8)	18 (50)	10 (41.7)	7 (38.8)	(53.8)	6 (50.0)
I am interested and willing to be involved in the data collection using these formats	Strongly disagree	0	0	0	0	0	0	0	0	0	0
	Disagree	0	0	0	0	0	0	0	0	0	6 (50.0)
	Neutral	0	0	0	0	0	3 (8.3)	0	4 (22.2)	0	1 (8.3)
	Agree	4 (25.0)	8 (88.9)	20 (40.0)	24 (53.3)	19 (52.8)	20 (55.6)	20 (83.3)	8 (44.4)	15 (57.7) 11	0
	Strongly agree	12 (75.0)	1 (11.1)	30 (60.0)	21 (46.7)	17 (47.2)	13 (36.1)	4 (16.7)	6 (33.3)	(42.3)	5 (41.7)
The data collection format is easy to understand	Strongly disagree	0	0	0	0	0	1 (2.8)	0	0	0	0
	Disagree	0	0	8 (16.0)	3 (6.7)	0	2 (5.6)	0	0	2 (8.0)	0
	Neutral	0	1 (11.1)	6 (12.0)	1 (2.2)	0	3 (8.3)	0	8 (44.4)	1 (4.0)	1 (8.3)
	Agree	12 (75.1)	4 (44.4)	26 (52.0)	29 (64.4)	27 (75)	10 (27.7)	17 (70.8)	7 (38.9)	18 (72.0)	7 (58.3)
	Strongly agree	4 (25.0)	4 (44.4)	10 (20.0)	12 (26.7)	9 (25)	20 (55.6)	7 (29.2)	3 (16.7)	4 (16.0)	4 (33.3)
The individual items appear relevant and useful	Strongly disagree	0	0	0	0	0	0	0	0	0	0
	Disagree	0	0	0	0	0	1 (2.8)	0	0	1 (3.9)	0
	Neutral	0	0	2 (4.0)	1 (2.2)	1 (2.8)	2 (5.6)	0	5 (27.8)	1 (3.9)	7 (58.3)
	Agree	1 (6.3)	5 (83.3)	25 (50.0)	28 (62.2)	22 (61.1)	22 (61.1)	19 (79.2)	10 (55.6)	22 (84.6)	1 (8.3)
	Strongly agree	15 (93.8)	1 (16.7)	23 (46.0)	16 (35.6)	13 (36.1)	11 (30.7)	5 (20.8)	3 (16.7)	2 (7.7)	4 (33.3)
I believe this form should be part of our normal work	Strongly disagree	0	0	0	0	0	0	0	0	0	0
	Disagree	1 (6.3)	2 (25.0)	0	0	2 (5.6)	0	0	1 (5.6)	0	1 (8.3)
	Neutral	0	0	0	0	0	4 (11.1)	1 (4.2)	2 (11.1)	1 (3.9)	2 (16.7)
	Agree	8 (50.0)	6 (75.0)	24 (48.0)	18 (40.0)	26 (72.2)	23 (63.9)	17 (70.8)	12 (66.7)	22 (84.6)	9 (75.0)
	Strongly agree	7 (43.8)	0	26 (52.0)	17 (60.0)	8 (22.2)	9 (25.0)	6 (25.0)	3 (16.7)	3 (11.5)	0
I feel confident using the data collection format	Strongly disagree	1 (6.3)	0	0	1 (2.2)	3 (8.3)	0	0	0	0	0
	Disagree	0	2 (22.2)	3 (6.0)	0	1 (2.8)	0	0	1 (5.6)	0	0
	Neutral	8 (50.0)	0	2 (4.0)	1 (2.2)	1 (2.8)	4 (11.1)	1 (4.2)	8 (44.4)	1 (3.9)	6 (50.0)
	Agree	7 (43.8)	4 (44.4)	34 (68.0)	24 (53.3)	20 (55.6)	22 (61.1)	18 (75.0)	5 (27.8)	20 (76.9)	1 (8.3)
	Strongly agree	0	3 (33.3)	11 (22.0)	19 (42.2)	11 (30.6)	10 (27.8)	5 (20.8)	4 (22.2)	5 (19.2)	5 (41.7)

Over time, there was an overall reduction of perceived utility. In 15 of the T1–T2 comparisons, there was a reduction of 20% or greater. These were especially seen in South Africa. In only one instance was there an increase of more than 20% in positive evaluation for an item from T1 to T2; this was for the level of confidence in using the formats in India. Finally, Nepal and Nigeria were the two sites with the most positive assessments of feasibility and acceptability by respondents (i.e. both scoring over 80% positive on 14/18 items).

## Discussion

A functioning routine mental health information system is required to guide the process of reducing the treatment gap for mental illness globally.^10^ The poor availability of data is a hindrance for the achievement of that goal in LMIC.^11^ A mental health information system aims to improve the effectiveness and efficiency of the mental health service and ensure more equitable delivery. Information that enables managers and service providers to make more informed decisions could aid the delivery of quality care.^12^ Given that most LMIC do not have any, or only very few, indicators to monitor mental healthcare as part of their existing Health Management Information System (HMIS), this study aimed to assess the performance and utility of a new set of indicators in five LMIC (India, Nepal, Nigeria, South Africa and Uganda). This is in line with WHO's Mental Health Action Plan 2013–2020, which sets a target of 80% of countries to routinely collect and report on a core set of mental health indicators within their national information systems by 2020.^13^ The new set of indicators go beyond monitoring of diagnoses, commonly the only indicator included in information systems in LMIC,^5^ to include subsequent need for, and utilisation of services, as well as related progress and cost.

Across countries we found that a newly developed and introduced set of indicators for routine monitoring of mental health service demonstrated good performance and high levels of perceived utility by non-specialist primary healthcare workers. First, we found high levels of completion (78% when considering all indicators) as well as high levels of accurate completion (87%). This was especially so for indicators on diagnosis (using multiple choice responses), severity of patients' problems and treatment provided. Documentation of functioning and follow-up also had good completion and correctness rates. Items capturing payment and referrals scored lower, even though this was mainly limited to one site (Uganda). Moreover, performance seemed to improve over time. These findings are important given the lack of studies examining the performance of indicators for monitoring mental health services. A systematic review of 106 publications on performance indicators for public mental healthcare found that only two studies explicitly assessed issues of data reliability, even though inadequate completeness and correctness are known risks for utility of data.^14^

Second, health workers' views about the feasibility of the routine conduct of mental healthcare monitoring using the new forms were generally positive across the five settings (and especially so in Nepal and Nigeria). The majority of measurements across countries and time points had more than 80% positive responses regarding the importance attached to them, the motivation and ease of their use, the usefulness of the collected information, the perceived need for the indicators and level of confidence in using them. Health workers were less positive about the additional time required for the completion of the forms. There was a trend of a downward drift in positive attitudes over time, though these were mostly observed in South Africa.

While the positive results were quite uniform across sites and time-points, the especially high levels of perceived feasibility and acceptability in the first month after training and introduction of the indicators suggests that ongoing training and supervision may contribute to keeping such positive attitudes. At the same time, reduction in positive attitudes over time did not result in reduced performance 6–9 months afterwards. On the contrary, continued use actually seemed to improve the results over time. The concern of health workers with regard to the time required for the additional reporting is understandable given the high workload in many of the understaffed health facilities in LMIC.^15^ It is nevertheless instructive that this concern did not seem to translate to poor performance or negative attitudes towards the importance or feasibility of implementing the surveillance system.

The set of indicators tested included those measuring needs, utilisation and outcomes. The combination of these would provide information necessary to assess coverage. Evaluating coverage of mental health programmes is essential in order to track efforts to scale up services for people with mental disorders.^16^ It is therefore reassuring that these indicators performed well in this study. The addition of an indicator for financial protection for mental health services would provide valuable data to help monitor the level of universal health coverage, an important goal within the United Nations Sustainable Development Goals. However, with only three countries (Nepal, Nigeria and Uganda) adopting the indicator in this study and with poorer performance compared with other indicators, it would appear that such an addition might be a step too far.

There are a number of limitations that should be considered in interpreting the findings of this study. First, the structured interviews with health workers might have introduced a bias towards social desirability, with a tendency for respondents to over-report use. Still, we saw low ratings on a number of questions, indicating that critical perspectives were also voiced. Second, not all countries used the same or the full set of indicator ratings. The variance between sites is a result of a process of in-country consultations and decision-making with regards to selection and formulation of the indicators. Third, this study did not include the assessment of (a) the quality of information that is reported using these new indicators (i.e. it did not include any validation of the completed information, for example, assessing the accuracy of the diagnosis given), or (b) the use of the information subsequent to collection (i.e. uptake within the larger HMIS system). The assessment of correctness of completion therefore only refers to whether and how the newly introduced formats were completed – a first threshold for adequate use in practice. Fourth, different countries utilised different selection methods for recruiting respondents (random, purposive and convenience), which may detract from the uniformity of the findings. Finally, this study focused exclusively on health facility level indicators directly related to service provision. Other health system indicators (e.g. number of trained personnel, availability of medications) are equally important for adequate monitoring of mental health services.^7^

This study has several implications. First, efforts to close the mental health treatment gap should be matched with efforts to close the information gap associated with mental healthcare in LMIC.^10^ This is especially pertinent, as meaningful planning for scaling up mental health services cannot take place in the absence of reliable mental health information to serve as a reliable benchmark of service utilisation and trends over time. Based on the results of this study, a limited set of indicators can be recommended for adoption into routine mental healthcare surveillance, which will be particularly useful when rolling out mhGAP-IG programmes.

Second, in addition to improved planning, the integration of a set of mental health indicators in HMIS will be an important ingredient for maintaining or improving the quality of care. Health workers and/or their supervisors, can use the information to better monitor the adequacy of initiated treatments following diagnosis, treatment adherence or drop-out, as well as patients' improvements over time, or lack thereof.

Third, if the set of indicators that were part of this study were integrated in HMIS and adequately implemented to collect data within routine primary healthcare settings, they could potentially be used to monitor coverage for mental healthcare through the following combination of indicators; *diagnosis*, (*change in*) *severity*, (*change in*) *functioning*, (*frequency of*) *treatment utilisation*, and also in low-resource health systems. This is especially salient as most mental health services information is limited to tertiary care settings^17^ and most focuses on health system inputs rather than processes or outcomes.^12^ Monitoring the level of financial protection may require a different method, for example, household surveys. Moreover, for calculation of actual coverage, reliable prevalence data are needed (i.e. the denominator), which may be unavailable in many settings.

Fourth, perceived utility is especially high shortly after training. Even though across the board introduction after training seems sufficient to achieve good performance, ongoing supervision and booster trainings may be needed to sustain positive attitudes towards use.^15^

Fifth, the next steps should involve the evaluation of using this set of indicators outside of a research programme. While the study took place in a routine care setting, a non-research setting will more closely reflect real-life implementation. Also, future research should evaluate the use of mental healthcare indicators within the HMIS process subsequent to data collection, i.e. processing, analyses, dissemination and use at facility levels, as well as beyond.

In conclusion, this study set out to explore the feasibility of monitoring mental healthcare when provided at scale by non-specialist health workers. The study demonstrates high levels of performance and utility across time for a set of indicators that could ultimately be used to monitor the coverage of mental healthcare in primary healthcare settings in five LMIC and contribute to maintaining or improving the quality of care. We recommend that these indicators be included in existing health information systems, and adopted as part of systems strengthening interventions necessary to facilitate the rolling out of the mhGAP-IG package, and to allow for evidence-based and data-driven decision-making and planning.

## Supplementary material

For supplementary material accompanying this paper visit http://dx.doi.org/10.1192/bjo.2019.22. click here to view supplementary material
