# Supplementary material for: Evaluation of performance and perceived utility of mental healthcare indicators in routine health information systems in five low- and middle-income countries
Source: BJPsych Open. 2019 Aug 6;5(5):e70. doi: 10.1192/bjo.2019.22 (PMC6688458; doi:10.1192/bjo.2019.22)
Supplement: Supplementary file 1 [file S205647241900022Xsup001.docx]

| **INSTRUCTION**: This data sheet is to be completed for each patient with a mental health diagnosis, and should be processed in line with instructions for HMIS (or other routine information system). |
| --- |

|  | | | | | | | | | | | | | | | | | | | | | | | | | | | | | | | | | | | | | | | | |
| --- | --- | --- | --- | --- | --- | --- | --- | --- | --- | --- | --- | --- | --- | --- | --- | --- | --- | --- | --- | --- | --- | --- | --- | --- | --- | --- | --- | --- | --- | --- | --- | --- | --- | --- | --- | --- | --- | --- | --- | --- |
| **DIAGNOSIS, SEVERITY & FUNCTIONING** | | | | | | | | | | | | | | | | | | | | | | | | | | | | | | | | | | | | | | | | |
|  | | | | | | | | | | | | | | | | | | | | | | | | | | | | | | | | | | | | | | |  |  |
| 1a. **Mental health diagnosis.** Circle the disorders that in your clinical judgment, the patient is suffering from, or circle the disorder for which the patient is currently under treatment. | | | | | | | | | | | | | | | | | | | | | | | | | | | | | | | | | | | | | | | | |
|  | **1** | | | Depression | | | | | | | | | **4** | | Epilepsy | | | | | | | | | | | **0** | |  | | | | | | | | | | | | |
|  | | | | | | | | | | | | | | | | | | | | | | | | | | | | | | | | | | | | | | | | |
|  | **2** | | | Alcohol Use Disorder | | | | | | | | | **5** | | Other (specify in box below) | | | | | | | | | | | **0** | |  | | | | | | | | | | | | |
|  | | | | | | | | | | | | | | | | | | | | | | | | | | | | | | | | | | | | | | | | |
|  | **3** | | | Psychosis (including schizophrenia,  manic depression/bipolar) | | | | | | | | |  | | | | | | | | | | | | | | | | | | | | | | | | | | | |
|  |  | | |  |  |  |  |  |  |  |  |  |  | | | | | | | | | | | | | | | | | | | | | | |  | | | | |
| 1b. **Specify exact diagnosis/substance used** | | | | | | | | | | | | |  |  |  |  |  |  |  |  |  |  |  |  |  |  |  |  |  |  |  |  |  |  |  |  |  |  |  |  |
| 1c. **Clinical Global Impression Severity (CGI-S).** Considering your total clinical experience with this particular population, how mentally ill is the patient at this time? Complete for all patients*.* | | | | | | | | | | | | | | | | | | | | | | | | | | | | | | | | | | | | | | | | |
|  | | | | | | | | | | | | | | | | | | | | | | | | | | | | | | | | | | | | | | | | |
|  | | *Normal, not at all ill* | | | | *Borderline mentally ill* | | | | | *Mildly ill* | | | *Moderately ill* | | | | | | *Markedly ill* | | | | | *Severely ill* | | | | | | *Among the most extremely ill* | | | | |  | | | | |
|  | |  |  |  |  |  |  |  |  |  |  |  |  |  |  |  |  |  |  |  |  |  |  |  |  |  |  |  |  |  |  |  |  |  |  |  | | | | |
|  | |  | | **1** |  |  | **2** |  | | |  | **3** |  |  | | **4** | |  | | |  | **5** | |  |  | | **6** | |  | |  | **7** | |  | |  | |  | | |
| 1d. **Assessment Functioning Scale** | | | | | | | | | | | | | | | | | | | | | | | | | | | | | | | | | | | | | | | | |
| In the past 30 days, for how many days were you totally unable to carry out your usual activities or work because of the health condition? | | | | | | | | | | | | | | | | | | | | | | | | | | | | | | | | |  | |  | |  | | |  |
| **TREATMENT** | | | | | | | | | | | | | | | | | | | | | | | | | | | | | | | | | | | | | | | | |
|  | | | | | | | | | | | | | | | | | | | | | | | | | | | | | | | | | | | | | | |  |  |
|  | | | | | | | | | | | | | | | | | | | | | | | | | | | | | | | | | | | | | | | | |
| 2a. **Interventions administered by clinician or aide.** Circle all that apply or “none.” | | | | | | | | | | | | | | | | | 2b. **Referred for other services.** Circle all that apply or “none.” | | | | | | | | | | | | | | | | | | | | | | | |
|  | | | **1** | Medication: specify | | | | | |  | | | | | | |  | | **1** | | | | Other medical services | | | | | | | | | | | | | | | | | |
|  | | | | | | | | | | | | | | | | | | | | | | | | | | | | | | | | | | | | | | | | |
|  | | | **2** | Psychoeducation | | | | | | | | | | | | |  | | **2** | | | | Psychiatrist | | | | | | | | | | | | | | | | | |
|  | | | | | | | | | | | | | | | | | | | | | | | | | | | | | | | | | | | | | | | | |
|  | | | **3** | Psychosocial support | | | | | | | | | | | | |  | | **3** | | | | Other non-medical services | | | | | | | | | | | | | | | | | |
|  | | | | | | | | | | | | | | | | | | | | | | | | | | | | | | | | | | | | | | | | |
|  | | | **4** | Other: Specify | | | | |  | | | | | | | |  | | **X** | | | | Others | | | | | | |  | | | | | | | | | | |
|  | | | | | | | | | | | | | | | | | | | | | | | | | | | | | | | | | | | | | | | | |
|  | | | **5** | None | | | | | | | | | | | | |  | | **5** | | | | None | | | | | | | | | | | | | | | | | |
|  | | | | | | | | | | | | | | | | | | | | | | | | | | | | | | | | | | | | | | | | |
| **Follow-up** | | | | | | | | | | | | | | | | | **COST** | | | | | | | | | | | | | | | | | | | | | | | |
|  | | | | | | | | | | | | | | | | |  | | | | | | | | | | | | | | | | | | | | | |  |  |
| 3. **Treatment follow-up**. Circle the option that indicates whether this was intake or (un)scheduled follow-up consultation. | | | | | | | | | | | | | | | | | **4a. Payment for consultation.** What does the patient need to pay for today’s consultation? [medical care only; do not include travel costs] | | | | | | | | | | | | | | | | | | | | | | | |
|  | | | **1** | First consultation | | | | | | | | | | | | |  | | **1** | | | | All of the costs: consultation + any medication, tests | | | | | | | | | | | | | | | | | |
| 2 | | | | | | | | | | | | | | | | |  | | | | | | | | | | | | | | | | | | | | | | | |
|  | | | **2** | Scheduled appointment | | | | | | | | | | | | |  | | **2** | | | | Some of the costs: e.g. co-payment/ fee, medication | | | | | | | | | | | | | | | | | |
|  | | | | | | | | | | | | | | | | |  | | | | | | | | | | | | | | | | | | | | | | | |
|  | | | **3** | Unscheduled: no appointment given | | | | | | | | | | | | |  | | **3** | | | | None of the costs: free at the point of use | | | | | | | | | | | | | | | | | |
|  | | | | | | | | | | | | | | | | |  | | | | | | | | | | | | | | | | | | | | | | | |
|  | | | **4** | Unscheduled: return after period of default | | | | | | | | | | | | | 4b. **Out of pocket costs.** If answer to 4a was ‘1’ or ‘2’, please indicate how much is to be paid: | | | | | | | | | | | | | | | | | | | | | | | |
|  | | | | | | | | | | | | | | | | |  |  |  |  |  |  |  |  |  |  |  |  |  |  |  |  |  |  |  |  |  |  |  |  |
|  | | | **5** | Unscheduled: forgot appointment | | | | | | | | | | | | |  | |  | | | | | | | | | | | | | | | | | | | | | |
